# Initial coral assemblage drives benthic community response to different disturbance type events

**DOI:** 10.1371/journal.pone.0317515

**Published:** 2025-05-29

**Authors:** Ahmyia J. Cacapit, Anela K. Akiona, Esmeralda A. Alcantar, Alex Araújo, Isa Bersamin, Ninoshka Betancourt, Daeyla’ Boyd, Bostony Braoudakis, Zachary Capilitan, Angelica Dimas, Theresa Duncan, Clinton B. Edwards, Audrey Ellias, Beverly J. French, Nahir Guadaloupe, Charles Hambley, Maxine Hauser, Nikki Jackson, Phi Lang, Mary Liesegang, Orion McCarthy, Nicole Pedersen, Elena Pilch, Samantha Scheibler, Rex Shettlesworth, Jackson Shoultz, Blake Stoner-Osborne, Brian J. Zgliczynski, Stuart A. Sandin

**Affiliations:** 1 Center for Marine Biodiversity and Conservation, Scripps Institution of Oceanography, UC San Diego, La Jolla, California, United States of America; 2 Department of Freshwater and Marine Ecology, University of Amsterdam, Amsterdam, Netherlands; 3 Department of Science and Technology, Antillean Adventist University, Mayagüez, Puerto Rico, United States of America; 4 Department of Biology, University of Guam, Mangilao, Guam, United States of America; 5 Department of Biology, Universidad Ana G. Mendez, Recinto de Cupey, San Juan, Puerto Rico, United States of America; 6 Department of Marine Science, California State University Monterey Bay, Monterey Bay, California, United States of America; 7 Department of Environmental Science and Biology, Eckerd College, St. Petersburg, Florida, United States of America; 8 Department of Geological Science, California State University Fullerton, Fullerton, California, United States of America; 9 Department of Biology, University of Portland, Portland, Oregon, United States of America; 10 Department of Natural Sciences, University of Puerto Rico, Humacao, Puerto Rico, United States of America; 11 Department of Biology, Georgia Southern University, Statesboro, Georgia, United States of America; 12 Department of Biology, Agnes Scott College, Decatur, Georgia, United States of America; 13 Department of Biology, Suffolk University, Boston, Massachusetts, United States of America; 14 Department of Biology, University of Southern California, Los Angeles, California, United States of America; 15 Department of Biology, Ball State University, Muncie, Indiana, United States of America; 16 Department of Biology, Abilene Christian University, Abilene, Texas, United States of America; 17 Department of Oceanography, University of Hawaii at Manoa, Honolulu, Hawaii, United States of America; Florida Atlantic University, UNITED STATES OF AMERICA

## Abstract

An increase in the intensity and frequency of extreme environmental conditions due to anthropogenic climate change impacts coral reefs through myriad stressors, from elevated sea-surface temperatures to increased storm activity. A reef’s response to these disturbances can be influenced by factors including taxonomic composition, life history strategies, or spatial patterning of the reef community members. We explored the disturbance-specific responses of coral reefs by following changes in benthic cover of major functional groups, community assemblage, and the response of common coral taxa at six islands across the central Pacific over the course of two years. We observed a decrease in average coral cover at four of the six islands, with differing underlying shifts in assemblage structure. Reefs with the highest pre-disturbance benthic coverage of *Montipora* spp. displayed significant increases in average hard coral cover compared to those where *Acropora* spp. was in the highest abundance; *Acropora*-dominated reefs showed significant declines in coral cover especially when exposed to the physical stress associated with a cyclone. Change in total coral cover was variable between islands within the same region, even among adjacent islands facing similar disturbance. These results highlight the importance of assemblage composition in influencing how benthic communities respond to major disturbance events such as thermal stress or cyclones. Improving our understanding of the drivers of differential community responses to disturbance will be important in predicting future changes in reef structure under changing ocean conditions.

## Introduction

On coral reefs, patterns of organismal abundance, diversity, and stability are intrinsically linked to different ecological disturbances [1,2]. Global warming driven by anthropogenic activity has caused an increase in global temperature, resulting in shifts in oceanographic conditions including the frequency and intensity of marine heat waves and storm events [3]. The continued increase in temperature is predicted to drive increases in the frequency and intensity of mass coral bleaching events [4]. While not immediately fatal, prolonged periods or recurring episodes of bleaching can lead to subsequent macroalgae overgrowth and death of corals, reducing both the diversity and productivity of reef-building organisms [5–9]. Hot-water-induced bleaching has further been linked to chronic physiological shifts including decreased population fitness and altered metabolic response [10]. Periods of intense heat stress can have major implications on coral communities, even shifting the dominant functional group from hard coral to macroalgae on reefs [11]. Local conditions including high macroalgal cover, low herbivorous fish populations, and poor water quality from sewage runoff can have additive pressures on reefs when impacted by larger disturbances [12,13].

Anthropogenically driven rglobal warming has also resulted in an increase in the frequency and magnitude of tropical cyclones [3,14]. These storms can cause mechanical damage to reef-building corals through fragmentation and colony dislodgement [15–18] and can suppress photosynthesis due to reduced light availability caused by sedimentation [16,19,20]. Assemblage shifts may occur if coral taxa are unable to return to their previous population structure following major disturbances, due in part to a dramatic loss of vulnerable functional coral groups.

Scleractinian corals are taxonomically and phenotypically diverse, employing a wide array of life history strategies that differentially respond to small-scale stressors and large-scale regional disturbance events. These strategies are often generalized based on a combination of characteristics including morphology, reproductive mode, generation time, growth rate, and symbiont composition [21]. Despite variation across species, coral genera broadly can be characterized as employing one of the following strategies: competitive, generalist, and stress-tolerant [22–24]. These life-history strategies\contextualize characteristics that influence how a group of organisms will react to both disturbance and stress, where extreme cases result in mass die-off. By applying a life-historical framework to coral reef systems, we can understand how major contributing coral genera may respond to wide-scale disturbance events and, as a result, how overall benthic cover may change following disturbance [23,25,26].

Specific physical and physiological characteristics, such as morphology, growth rates, and fecundity, determine how a species responds to various disturbance events. On many Indo-Pacific coral reefs, upright, branching coral species (e.g., *Acropora* spp.) are considered to have a low tolerance to disturbance due to their physical and physiological vulnerability to bleaching and fragmentation [27–29]. In some regions, mass Acroporid decline was induced by thermal stress and shifted the ecological functioning of reefs where this genus was the predominant taxa [30,31]. However, these species can have high reproductive outputs via the survival and growth of clonal fragments and sexually derived recruits [15,31]. In contrast, morphologically robust corals with an extensive foundation, such as massive or encrusting species (e.g., *Montipora* spp. and *Porites* spp.), can withstand damage from physical disturbance more readily due to their stability through larger benthic contact [32] and are less susceptible to bleaching than other genera due to mass transfer theory [33]. However, these corals often exhibit slower growth rates in comparison to more competitive groups [34]. There are also exceptions to the stress tolerance of these taxa; intense heat events exceeding a region’s historical thermal history can induce critical bleaching in *Montipora* spp. populations even in unpopulated regions [35–37].

Changes in community composition on a reef can reflect the history of disturbances that the region has experienced [38]. Here, we examine the change in benthic percent cover of major functional groups, including a more targeted consideration of change among four common taxa of stony corals, *Acropora* spp., *Porites* spp., *Montipora* spp., and *Pocillopora* spp., at six islands in the central Pacific to describe patterns of changing benthic percent cover in response to distinct disturbance events. The functional groups and coral genera were chosen as focal groups for this study because they comprised majority of the total benthic composition at the study islands. We hypothesize that forereefs dominated by low-tolerant taxa will show drastic declines in coral cover whereas reefs primarily composed of stress-tolerant taxa will have stable coral cover when faced with similar disturbances. We show how coral taxa display differential responses when faced with two bleaching events, a storm event, and a period of relative stability, and how these responses may be linked with different life history strategies of benthic taxa.

## Methods

**Fig 1:**
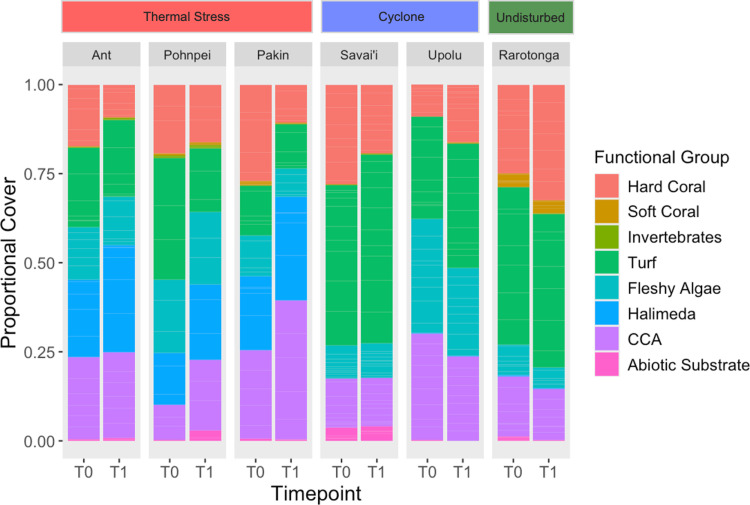
Proportional cover of major benthic functional groups at each survey island by timepoint. The major benthic functional groups were chosen based on distinct characteristics and ecological services that each group provides including hard coral, soft coral, invertebrates, turf, fleshy algae, *Halimeda*, crustose coralline algae (CCA), and abiotic substrate.

We quantified the benthic community composition of reef habitats at six islands across the central Pacific (Fig.1) over a two-year time period. The number of sites surveyed per island ranged from 3-10 sites (Table 1, S1 Table). Three islands of the Federated States of Micronesia (FSM), Pohnpei, Ant Atoll, and Pakin Atoll, were surveyed in August of 2016 (*t*_*0*_) and 2018 (*t*_*1*_). In 2016, a strong El Nino event occurred in FSM, elevating sea surface temperatures and causing two mass bleaching events (Table 1) [40,41]. Coral reefs in Samoa were surveyed in December 2017 (*t*_*0*_) and 2019 (*t*_*1*_) on the islands of Savai’i and Upolu. In 2018 between sampling periods, Cyclone Gita passed along the southwest coast region of both islands (Table 1). Finally, benthic communities were surveyed at Rarotonga in the Cook Islands, in January of 2018 (*t*_*0*_) and November of 2019 (*t*_*1*_). During the survey period, Rarotonga was not affected by any major disturbance events, serving as a stable interval for this study (Table 1).

**Table 1:** The data characteristics of each survey island. Photoquadrat data collected at each island along with disturbance events occurring at each island between survey periods. Degree Heating Week (DHW) is a reliable predictor for regional bleaching events, with bleaching occurring above 4 °C-weeks and mass mortality expected above 8 °C-weeks [39].

Island	Survey Year	Number of Sites	Photos Per Site	Disturbance Type	Disturbance Date	Disturbance Intensity
Ant Atoll	2016	6	25-40	Thermal stress	August 2016; November 2016	4°C-weeks; 12°C-weeks
Ant Atoll	2018	6	26-40			
Pakin Atoll	2016	5	28-38			
Pakin Atoll	2018	5	26-40			
Pohnpei	2016	3	33-36			
Pohnpei	2018	3	38-40			
Rarotonga	2018	7	10	NA	NA	NA
Rarotonga	2019	7	10			
Savai’i	2017	10	16-18	Cyclone	February 2018	Category 3 Storm
Savai’i	2019	10	16-18			
Upolu	2017	9	18			
Upolu	2019	9	18			

All data were collected using photoquadrat surveys conducted on the forereefs at a depth of 10-12 m with three 25m transects per site. Sites were established at semi-random locations. Photos were captured at 2m intervals along the transects for a total of 10-40 images per site. Photoquadrat images were collected with a monopod positioned perpendicular to the plane of the sea surface capturing an area of approximately 0.9 m x 0.6 m of the benthos.

Data from Savai’i, Upolu, and Rarotonga were collected under Agreement ref.: PM/CRIOBE/SAMOA/13 granted by the Samoa Fisheries: Ministry of Agriculture and Fisheries. Data from Ant Atoll, Pakin Atoll, and Pohnpei were collected under scientific research permits in collaboration with the Conservation Society of Pohnpei and OneReef.

### Benthic community assessment

The relative abundance of the sessile, benthic community was estimated using CoralNet, an analysis tool facilitating benthic image annotation. Point-based annotations were completed for each of the 100 points that were randomly stratified across each image. The benthic substrate or organism located under each point was manually identified to the finest taxonomic resolution possible (i.e., to species level, if possible from imagery, or to genus level). For analyses of coarse composition, major benthic functional groups we defined to include hard coral, soft coral, invertebrates, turf, fleshy algae, *Halimeda*, crustose coralline algae (referred to as ‘CCA’), and abiotic substrate (e.g., sand, rubble, etc). For subsequent analyses, coverage data of corals were collapsed into five groups. Four cosmopolitan taxa were identified as focal taxa because they contributed to the highest coral cover: *Acropora* spp., *Montipora* spp., *Pocillopora* spp., and *Porites* spp., where all other coral taxa were categorized as ‘other’. The four focal taxa displayed a gradient of proposed life history strategies as well as common and ubiquitous distribution throughout the Pacific. Further, genus-level groupings provided robust categories as the common species within these genera share similar life history characteristics and similar functional framework [26,39,42,43]. Benthic compositional data were averaged across photographs for each site, with site as the independent replicate, and reported as percent benthic cover.

### Statistical analysis

To compare major functional group assemblage between islands, a PERMANOVA was performed with ‘timepoint’ and ‘island’ as fixed factors. The Euclidean distances of the community structure at each site was used for the PERMANOVA. The major benthic functional groups include hard coral, soft coral, turf, fleshy algae, CCA, *Halimeda*, and abiotic substrate. Multivariate community structure was visualized using a non-metric multidimensional scaling (NMDS). Paired t-tests were used individually for each island to determine if there were significant differences between the percent average coral cover in *t*_*0*_ and *t*_*1*_. A one-factor analysis of variance (ANOVA) was used to determine the differences in percent change across islands, using sites as replicates for each island. A Tukey post-hoc test was conducted to determine which island combinations were statistically different from each other (p < 0.05).

## Results

In *t*_*0*_, coverage of hard coral across all six islands averaged 21.0 ±  3.0% (mean ±  standard error; SE) of the benthic community and decreased to an average of 17.4 ±  3.4% SE by the second survey period. The islands in Micronesia – Ant Atoll, Pakin Atoll, and Pohnpei -- experienced two thermal stress events between survey years (Table 1). Savai’i and Upolu, in Samoa, were affected by Cyclone Gita, a Category 3 Storm, between survey years (Table 1). No major disturbance event occurred at Rarotonga (Cook Islands) during the study period (Table 1).

The map shows survey islands and the geopolitical national to which they belong. Survey islands were chosen based on available survey data that spanned a two-year time interval. Disturbed islands were subjected to either a cyclone event or a thermal stress between survey periods whereas Rarotonga in the Cook Islands faced no major regional disturbance.

Based on a two-factor PERMANOVA, ‘island’ had a significant effect on the average functional group cover under 999 permutations (F = 4.13, p < 0.001). In Ant, Pakin, and Rarotonga, no benthic group composed > 30% of the benthos (Fig 1). *Halimeda* was the most prominent in the group in the survey islands within FSM and ranged from 14.5-22.8% in *t*_*0*_ as opposed to rela*t*ive absence seen in the 3 other islands. Turf algae was the most prominent benthic group in both timepoints in Savai’i, with 53.0% benthic cover by *t*_*1*_ (Fig 1).

**Fig 2:**
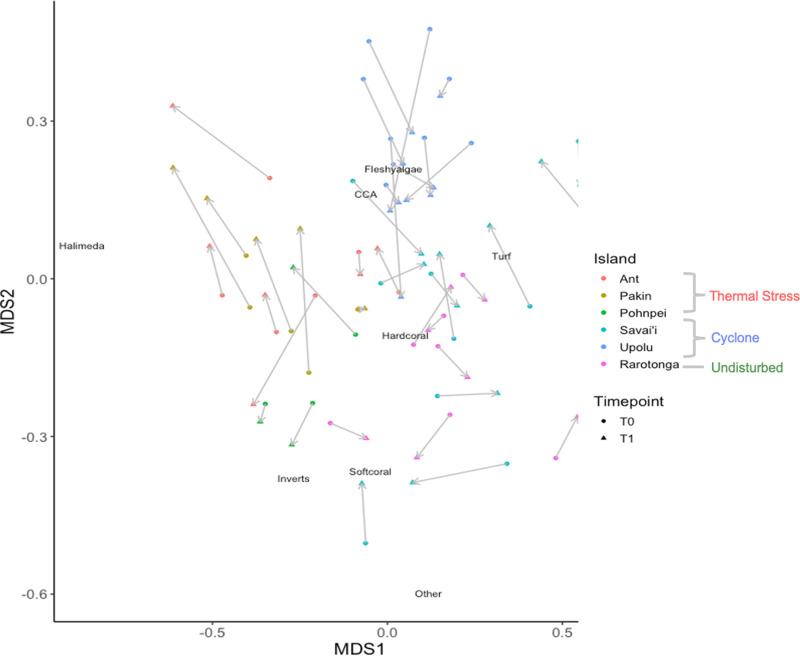
Non-metric multidimensional scaling (nMDS) of major benthic functional groups at each site in both timepoints (2D stress =  0.20). The plot was prepared using Bray-Curtis similarity and site scores are shown as points in ordination space. Arrows (grey) show the directionality of the same site between each timepoint to visualize how each site shifted from the first survey period to the next. Functional group scores on the plot are the weighted averages of the site scores and indicate the position of each group relative to the survey sites in the ordination space. ‘Island’ was found to have a significant effect on the relative similarity, as determined from a PERMANOVA (p = 0.001).

Based on a PERMANOVA, ‘Island’ has a significant effect on major functional group cover whereas ‘time’ did not based on 999 permutations (F = 8.80, p = 0.001) (Fig 1 and 2). Sites clustered based on island location. Clustering is seen in the NMDS from Upolu sites around ‘fleshy algae’ and from Rarotonga sites around ‘hard coral’ (Fig 2).

**Fig 3:**
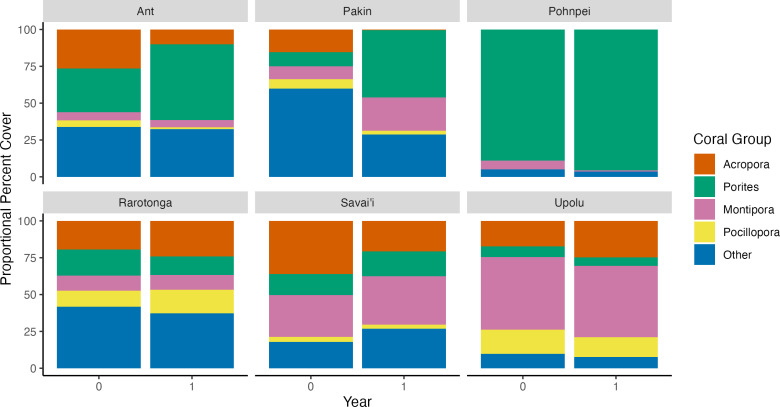
Proportional percent cover of major coral taxa by island during each survey year. The four focal taxa include *Acropora* spp., *Porites* spp., *Montipora* spp., and *Pocillopora* spp..

**Fig 4:**
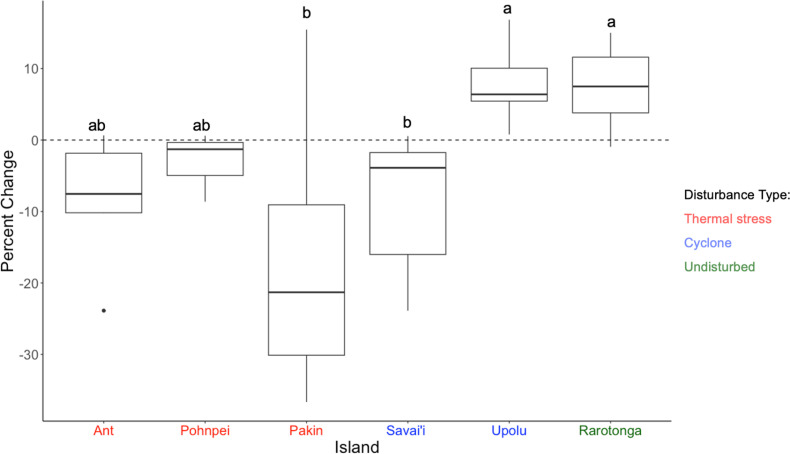
Percent change of island based on the average coral cover found in *t*_*0*_ vs.

To determine community assemblage shifts, we investigated changes in relative abundance by the four main coral genera found at each island. Upolu, Savai’i, and Rarotonga experienced significant changes in percent coral cover between the survey years based on paired t-tests (p = 0.0019, p = 0.017, p = 0.017, respectively). Coral cover at Savai’i decreased from 28.0 ±  6.2% SE to 19.4 ±  4.0% SE while coral cover at Upolu and Rarotonga increased from 8.9 ±  2.6% SE to 16.2 ±  3.4% SE and 25.0 ±  3.2% SE to 32.5% ±  2.1% SE, respectively (Fig 1). The genus with the highest percent cover on each island comprised 15-88% of the benthos and consisted of one of the following taxa: *Porites* spp., *Acropora* spp., and *Montipora* spp. (Fig 3). Change in percent coral cover was significant between islands based on a one-factor ANOVA (F = 6.37, p < 0.001). A Tukey post-hoc analysis showed significant differences in group means between the following island combinations: Pakin and Rarotonga, Pakin and Upolu, Savai’i and Upolu, Savai’i and Rarotonga (Fig 4).

contributed to the highest proportional cover of hard coral in the survey islands.

***t***_***1***_**. An** ANOVA and Tukey-post hoc tests were conducted to test for differences in percent hard coral cover. Islands with different letter assignments indicate island-combinations that significantly differed from each other include: Pakin and Rarotonga, Pakin and Upolu, Savai’i and Upolu, Savai’i and Rarotonga (p < 0.05).

### *Acropora* spp

**Fig 5:**
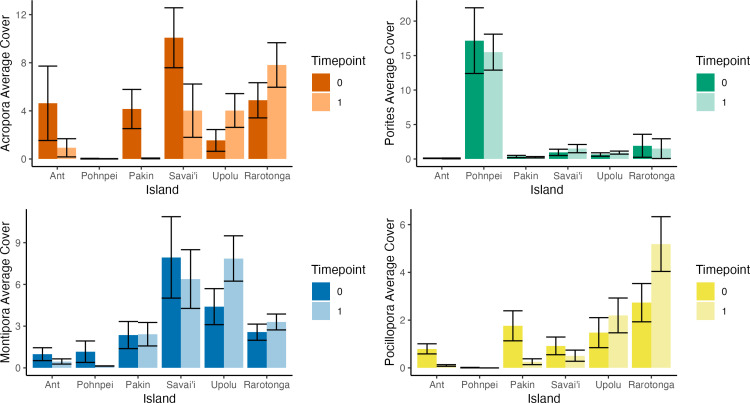
Percent average hard coral cover of dominant taxa during both survey periods at each island. Error bars represent standard error. Survey periods spanned two years of change.

In *t*_*0*_, *Acropora* spp. was the dominant taxon at three of the six survey islands and had the second highest average cover across all survey islands 13.4% (Fig 3). In Upolu and Rarotonga, *Acropora* spp. average cover increased significantly over the two survey periods (p = 0.024 and p = 0.039, respectively) (Fig 5) and proportional cover increased by 7.5% and 4.5%, respectively (Fig 4). Significant loss of *Acropora* spp. was observed at Savai’i (p = 0.041) (Figs 4 and 5). In FSM, *Acropora* spp. cover was 4.6% in Ant Atoll, 4.2% in Pakin Atoll, and 0.03% in Pohnpei in *t*_*0*_ (Fig 5); but by *t*_*1*_, *Acropora* spp. cover was recorded at less than 1% at each of these islands surveyed in FSM (Fig 5). In Upolu, *Acropora* spp. cover was 1.5% in *t*_*0*_ and 4.0% in *t*_*1*_.

### *Porites* spp

On average, *Porites* spp. had the highest percent cover across survey islands in *t*_*0*_ and *t*_*1*_. In *t*_*0*_, Ant Atoll and Pohnpei were the only islands where the genus was dominant. *Porites* spp. average cover did not change significantly at any island between the survey periods (Fig 5). *Porites* spp. cover in *t*_*0*_ at Ant Atoll, Pakin Atoll, and Pohnpei was 5.2%, 2.6%, and 17.2%, changing in *t*_*1*_ to 4.8%, 4.9%, and 15.5%, respectively (Fig 5). In Savai’i, *Porites* spp. cover was 4.0% in *t*_*0*_ and 3.3% in *t*_*1*_. In Upolu, *Porites* spp. cover was less than 1% in both time points. Proportional cover on Rarotonga and Upolu was 5.0% and 1.6% respectively (Fig 5).

### *Montipora* spp. and *Pocillopora* spp.

*Montipora* spp. cover change varied by island but was relatively stable over time, with an average percent change of 0.2%. *Montipora* spp. was dominant at Upolu in *t*_*0*_ whereas it was the second most dominant coral in Savai’i in *t*_*0*_ (Fig 4). In Rarotonga, Ant Atoll, Pakin Atoll, and Pohnpei, *Montipora* spp. cover ranged from 1.0% to 2.6%. *Pocillopora* spp. had the lowest initial average percent cover (1.3%) out of the four focal species across all islands, followed by *Montipora* spp. (3.2%) (Fig 5).

## Discussion

In this study, we examined trends in four focal taxa to illustrate shifts in coral assemblages at six different islands: three experiencing thermal stress, two experiencing cyclones, and one not experiencing any major disturbance.

In the FSM, Ant, Pakin, and Pohnpei did not undergo drastic shifts in community composition, despite experiencing thermal stress. These islands’ benthic communities all had relatively similar contributions, with no major functional group dominating each island at either time points (Fig 1). In contrast, turf composed the highest proportional cover of the benthos at Rarotonga, Savai’i, and Upolu. Rarotonga reefs have been documented with > 50% turf cover in previous decades [44]. Higher cover of fleshy algae was observed in Upolu than in Savai’i. However, we did not detect macroalgal outcompeting hard coral unlike previous studies which have seen rapid succession phase shifts within a year’s span at Upolu [45]. Macroalgae can influence benthic assemblage through direct interaction or indirect pathways, with varying degrees of severity on corals [9].

In islands facing similar disturbance events, we saw varying degrees of change, with the pattern of change linked to the initial taxonomic composition of the coral assemblage (Fig 3 and 5). Reef assemblages dominated by taxa such as *Montipora* spp. showed capability to resist rapid decline, for example the total coral cover in Upolu doubled in the months following a storm disturbance. In contrast, reefs primarily composed of *Acropora* spp. had lower capacity for coral cover growth, as seen with significant coral cover decline in Savai’i. Despite this observation, stability can potentially occur as seen in the Caribbean with lower coral cover [46].

Three of the focal islands experienced thermal stress over the study interval. Of these, the two reefs composed of *Porites* spp., Pohnpei and Ant Atoll, experienced a smaller degree of percent cover change relative to the reefs dominated by *Acropora* spp., Pakin Atoll. However, the changes observed in percent hard coral cover at the islands facing the thermal stress were not significant. Rarotonga, where no disturbance occurred within the survey period, served as a baseline for continuous change under no major regional stressor. Rarotonga’s reefs were dominated by *Acropora* spp. and showed significant increases in coral cover.

Three of the islands surveyed experienced thermal stress event during the study interval (Ant, Pakin, and Pohnpei in FSM), and no statistically significant changes in total coral cover were recorded from any of the islands. There were, however, notable shifts in the relative abundance of the coral taxa, where the shifts were related to the taxonomic differences in the initial coral assemblages (*t*_*0*_) (Fig 4). An increased frequency of thermal stress will likely lead to continued shifts in taxonomic structure, reflecting life history strategy of the affected taxa. For example, the highly variable *Acropora* spp. may be replaced by the more stress-tolerant *Porites* spp. like the shifts observed in Ant and Pakin Atoll in this study [47,48]. *Acropora* spp. has a well-documented vulnerability to coral bleaching [35,49–51]. Although overall change in percent cover was not significant in these islands, we still observed loss of *Acropora* spp. cover in Ant and Pakin (Figs 4 and 5). This change may transform a reef’s biological, ecological, and structural characteristics from its prior state [52].

Assemblage shifts were observed for the islands which experienced a cyclone event during the study period. Reefs assemblages in this study dominated by taxa such as *Montipora* spp. displayed higher resistance to disturbance. *Montipora* spp has exhibited relative stability similar to other regions like Okinawa [53]. In contrast to mass decline from bleaching stress seen in other regions, *Montipora* spp. observed in Upolu for this study exhibited relative resistance to mechanical stress [36,37]. Cyclone Gita’s path eastward, likely contributed the differing responses to the storm despite the proximity between the two islands. Moreover, the total hard coral cover at Upolu doubled from *t*_*0*_ to *t*_*1*_. Additionally, Savai’i, whose dominant assemblage was *Acropora* spp. saw a significant decrease in coral cover largely driven by the loss of *Acropora* spp., however, at Upolu, the opposite was observed. *Acropora* spp. has a range of growth forms including tabular, arborescent, corymbose, digitate, and massive. These morphological differences result in varying responses to wave intensity and the degree of change depending on colony-specific characteristics [33]. In addition, major decline of *Acropora* spp*.* has been observed from various stress types including storm activity and thermal stress [28,30]. Local conditions and bio-geophysical factors, such as highly sheltered sites, can influence the response in cover change like those observed between Savai’i and Upolu for *Acropora* spp. [28].

During imagery collection, it was noted that corals observed at Upolu were primarily juvenile corals, which may have been a contributing factor in different responses to the storm event between islands, as juvenile corals can have an increased capacity for regrowth after disturbance, relative to adult colonies [54–56].

Rarotonga, the only island which did not experience a disturbance event over the study period, showed the highest increase in overall coral cover change of all survey islands (Figs 1 and 3). By the second timepoint, over 30% hard coral cover was observed at this island, which has not been recorded since 1994 (44). Increases were observed in both *Acropora* spp. and *Pocillopora* spp., both fast-growing, competitive corals, which show to utilize resources more effectively in stable environments [23]. In contrast, *Porites* spp. decreased in percent coral cover likely due to other taxa outcompeting the group for space [24,57]. *Acropora* spp. has been observed to effectively maximize their growth capacity than more slow-growing taxa, recolonizing spaces both in disturbed and stable environments [58,59]. These results align with the assumptions of coral life history theory of continuous growth in the absence of disturbance [23,24].

Of the four focal genera, *Acropora* spp. exhibited the greatest variability, showing both significant decreases and small increases in percent cover. *Acropora* spp. are highly susceptible to both disturbance and disease but retain high growth rates, highlighting the boom-and-bust dynamics that this competitive genus can undergo from regional changes in the system [21,24,60,61]. Because *Acropora* spp. are disproportionately affected by thermal stressors and other environmental impacts, quantifying their percent cover over a small temporal scale can mask key insights into their recovery such as colony-level dynamics of fragment regrowth [62]. Short-term shifts can be observed from one year to the next; however, if given a stable period, *Acropora* spp. can maintain reef cover like surveyed reefs seen in Rarotonga. These observations highlight the importance of understanding taxonomic composition, including details of taxon-specific life history characteristics, to understand disturbance response.

Coral assemblages have shown a high capacity to recover from multiple disturbances, but differences in disturbance response affect taxon-specific resilience and have resulted in changes in composition following disturbances [63]. Changes from fast-growing, low-tolerance corals like *Acropora* spp., to slow-growing, resistant massive corals, such as *Porites* spp., highlight major losses in reefs that are reliant on susceptible groups for overall coral cover. The resulting shift to massive, more resilient corals decreases structural complexity which may impact diversity of both corals and reef fish [64,65] but also leaves behind a community dominated by stress-tolerant or generalist corals, one that perhaps will exhibit more stability when faced with disturbance events in the future. Differences in reef response can occur based on disturbance type, with thermal stress often having more prominent effects than other stressors like hurricanes and disease [66]. To better understand the impacts of disturbance driven changes, it is essential to investigate the patterns of long-term response on species composition following disturbance events. This study illustrates the dynamic patterns of coral reef response following major disturbances and highlights variability by island, even among islands within similar geographies.

## Supporting information

S1 Table Locations of study sites at each survey island. The table shows the site coordinates for all survey islands and their respective geopolitical affiliation. Each site was revisited after approximately 2 years.
(DOCX)
