# Supplementary material for: Initial coral assemblage drives benthic community response to different disturbance type events
Source: PLoS One. 2025 May 29;20(5):e0317515. doi: 10.1371/journal.pone.0317515 (PMC12122029; doi:10.1371/journal.pone.0317515)
Supplement: S1 Table — The table shows the site coordinates for all survey islands and their respective geopolitical affiliation. Each site was revisited after approximately 2 years. (DOCX) [file pone.0317515.s001.docx]

**S1 Table. Locations of study sites at each survey island.** The table shows the site coordinates for all survey islands and their respective geopolitical affiliation. Each site was revisited after approximately 2 years.

| Geopolitical Affiliation | Island | Site | Latitude | Longitude |
| --- | --- | --- | --- | --- |
| Federated States of Micronesia | Ant | 2 | 6.820 | 157.923 |
|  |  | 4 | 6.811 | 157.969 |
|  |  | 6 | 6.800 | 158.016 |
|  |  | 12 | 6.744 | 157.957 |
|  |  | 16 | 6.775 | 157.910 |
|  |  | 17 | 6.827 | 157.999 |
|  | Pohnpei | 6 | 6.876 | 158.103 |
|  |  | 7 | 6.837 | 158.112 |
|  |  | 8 | 6.800 | 158.112 |
|  | Pakin | 4 | 7.074 | 157.812 |
|  |  | 5 | 7.083 | 157.791 |
|  |  | 6 | 7.089 | 157.767 |
|  |  | 7 | 7.067 | 157.773 |
|  |  | 9 | 7.051 | 157.833 |
| Samoa | Savaii | 12 | -13.478 | -172.550 |
|  |  | 13 | -13.484 | -172.569 |
|  |  | 15 | -13.492 | -172.603 |
|  |  | 16 | -13.498 | -172.624 |
|  |  | 17 | -13.500 | -172.645 |
|  |  | 18 | -13.499 | -172.661 |
|  |  | 20 | -13.515 | -172.697 |
|  |  | 21 | -13.512 | -172.719 |
|  |  | 22 | -13.502 | -172.735 |
|  |  | 23 | -13.492 | -172.762 |
|  | Upolu | 1 | -13.799 | -171.779 |
|  |  | 2 | -13.794 | -171.796 |
|  |  | 3 | -13.780 | -171.802 |
|  |  | 5 | -13.772 | -171.838 |
|  |  | 6 | -13.774 | -171.857 |
|  |  | 7 | -13.768 | -171.873 |
|  |  | 12 | -13.793 | -171.948 |
|  |  | 13 | -13.804 | -171.975 |
|  |  | 14 | -13.803 | -171.997 |
| Rarotonga | Rarotonga | 1 | -21.219 | -159.835 |
|  |  | 3 | -21.200 | -159.786 |
|  |  | 4 | -21.202 | -159.777 |
|  |  | 6 | -21.211 | -159.739 |
|  |  | 7 | -21.247 | -159.721 |
|  |  | 8 | -21.272 | -159.732 |
|  |  | 11 | -21.245 | -159.831 |
